# Exploring the Impact of the Microbiome on Neuroactive Steroid Levels in Germ-Free Animals

**DOI:** 10.3390/ijms222212551

**Published:** 2021-11-21

**Authors:** Silvia Diviccaro, Valentina Caputi, Lucia Cioffi, Silvia Giatti, Joshua M. Lyte, Donatella Caruso, Siobhain M. O’Mahony, Roberto Cosimo Melcangi

**Affiliations:** 1 Dipartimento di Scienze Farmacologiche e Biomolecolari, Università degli Studi di Milano, Via Balzaretti 9, 20133 Milano, Italy; silvia.diviccaro@unimi.it (S.D.); lucia.cioffi@unimi.it (L.C.); silvia.giatti@unimi.it (S.G.); donatella.caruso@unimi.it (D.C.); 2 APC Microbiome Ireland, University College Cork, T12 YT20 Cork, Ireland; vcaputi@uark.edu (V.C.); joshua.lyte@usda.gov (J.M.L.); SOMahony@ucc.ie (S.M.O.); 3 Poultry Production and Product Safety Research Unit, United States Department of Agriculture, Agricultural Research Service, Fayetteville, AR 72701, USA; 4 Department of Anatomy and Neuroscience, University College Cork, T12 ND89 Cork, Ireland

**Keywords:** liquid chromatography–tandem mass spectrometry, hippocampus, cerebellum, cerebral cortex, hypothalamus, plasma, androgens, allopregnanolone

## Abstract

Steroid hormones are essential biomolecules for human physiology as they modulate the endocrine system, nervous function and behaviour. Recent studies have shown that the gut microbiota is directly involved in the production and metabolism of steroid hormones in the periphery. However, the influence of the gut microbiota on levels of steroids acting and present in the brain (i.e., neuroactive steroids) is not fully understood. Therefore, using liquid chromatography–tandem mass spectrometry, we assessed the levels of several neuroactive steroids in various brain areas and the plasma of germ-free (GF) male mice and conventionally colonized controls. The data obtained indicate an increase in allopregnanolone levels associated with a decrease in those of 5α-androstane-3α, 17β-diol (3α-diol) in the plasma of GF mice. Moreover, an increase of dihydroprogesterone and isoallopregnanolone in the hippocampus, cerebellum, and cerebral cortex was also reported. Changes in dihydrotestosterone and 3α-diol levels were also observed in the hippocampus of GF mice. In addition, an increase in dehydroepiandrosterone was associated with a decrease in testosterone levels in the hypothalamus of GF mice. Our findings suggest that the absence of microbes affects the neuroactive steroids in the periphery and the brain, supporting the evidence of a microbiota-mediated modulation of neuroendocrine pathways involved in preserving host brain functioning.

## 1. Introduction

Information relating to the chemical composition of ingested food, the dynamic equilibrium of the microbial ecosystem and the physiological state of the gastrointestinal (GI) tract reaches the brain through the so-called microbiome–gut–brain axis (MGBA). This is a complex bi-directional set of neuronal, immune, endocrine signaling pathways and molecules allowing communication between the gastrointestinal tract and the central nervous system (CNS) [1]. The gut microbiota is a key orchestrator of gut–brain axis communication, as besides maintaining the intestinal epithelial barrier integrity and providing information to the immune system, gut microbes are able to produce a number of neurotransmitters as well as microbial-derived neuroactive products such as short-chain fatty acids, and to directly signal the peripheral and central nervous systems, and potentially modulate their function [2,3]. In the brain, physiological processes such as the stress response, blood circulation, and digestion as well as tissue functioning, cell proliferation, and organ maturation are finely tuned by the combined action of the nervous and the endocrine systems, which utilize neurotransmitters and steroid hormones to exchange information among the different brain regions and with the peripheral organs [4]. Recent studies have shown that the gut microbiota is capable of influencing the production of glucocorticoid hormones from adrenal glands [1,4,5]. Additionally, a role of sex steroids synthesized from the gonads (i.e., androgens, estrogens and progesterone) has also been ascertained. Indeed, sex differences have been reported in the composition of the gut microbiota, with specific phyla, families and genera variances occurring with clear effects of gonadectomy and steroid hormone replacement on gut bacteria in rodents [6,7,8,9,10,11,12,13,14,15]. Furthermore, behaviour usually linked to estrous cycle stage such as visceral pain is not evident in germ-free (GF) mice [13], hence an appropriate microbiota colonization is necessary for appropriate functioning of the endocrine and nervous systems. This interaction of steroid hormones and the microbiome is also evident in human studies where sex differences have been noted in gut microbiota composition and diversity [14,15,16]. The gut microbiota itself also influences sex steroid levels both in rodents and humans [17,18,19,20]. Both human and animal studies highlight the reciprocal connections between sex steroid hormones (i.e., steroids synthesized by peripheral glands) and gut microbiota [6,7,8,9,10,11]. Interestingly, sex steroids are not only synthesized by gonadal glands but also within the nervous system (i.e., neurosteroids). Indeed, precursors of these steroids, such as pregnenolone (PREG) and dehydroepiandrosterone (DHEA), as well as the sex steroids themselves, such as progesterone (PROG), testosterone (T) and their metabolites, dihydroprogesterone (DHP), allopregnanolone (ALLO), isoallopregnanolone (ISOALLO) and dihydrotestosterone (DHT), 5α-androstane-3α, 17β-diol (3α-diol) and 17beta-estradiol (17β-E) have been identified in different brain regions [21,22]. Both steroid hormones and neurosteroids, which are both found in the nervous system, are collectively referred to as neuroactive steroids and are important physiological regulators of nervous system functioning [23]. In particular, DHP controls reproductive functions, as well as glutamatergic and GABAergic neurotransmission [24], whereas isoallopregnanolone influences the lipid bilayer model system containing cholesterol [25,26]. In the brain, androgen molecules have been shown to regulate dendritic spine maturation [27,28], behaviour [29,30], neurite growth [31], neurogenesis and neuronal survival [32], apoptosis [33] and catecholamine production [34]. To date, only one study, performed in male animals, has focused on the possible influence of the gut microbiota on neuroactive steroid levels. Indeed, as recently observed, specific pathogen-free (SPF) male mice displayed differential levels of neuroactive steroids in specific brain areas [35]. Nevertheless, GF animals, which are born and raised without any microorganisms, represent an invaluable tool for understanding the role of microbiota in modulating brain development and behaviour via the gut–brain axis [1,36,37,38]. Therefore, the current study investigates, for the first time, the impact of a complete lack of microbiome on the concentration of neuroactive steroids in both the periphery and the central nervous system of GF male mice [36], setting the base for understanding the biomolecular mechanisms behind the microbial-dependent modulation of the neuroendocrine system, with important implications for brain function and behavioral phenotypes.

## 2. Results

The levels of different neuroactive steroids were assessed in the plasma and different brain regions of male GF mice and compared with those observed in conventionally colonized (CV) mice. Quantitative analysis of all steroids was achieved based on calibration curves; each steroid concentration was calculated as pg/sample. Total pg/sample values were normalized by tissue weight (i.e., μL for plasma and mg for hippocampus, cerebellum, cerebral cortex and hypothalamus).

### 2.1. Assessment of Neuroactive Steroid Levels in Plasma

**Figure 1:**
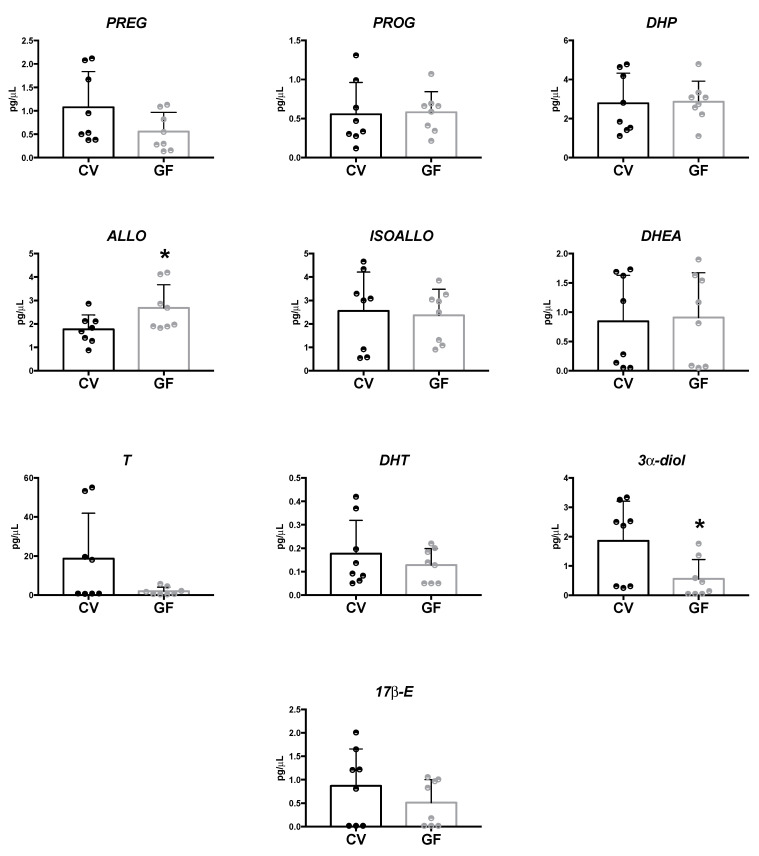
Levels of neuroactive steroids in the plasma of germ-free (GF) and conventional (CV) male mice. Data are expressed as pg/μL ± SD, n = 8 for each group. Unpaired Student’s *t*-test analysis: * *p* < 0.05 vs. CV mice.

LC–MS/MS analysis showed a significant increase in ALLO concentrations (*p* = 0.043), associated with a decrease in the levels of 3α-diol *(p =* 0.028) in the plasma of GF in comparison to CV mice (Figure 1). No significant changes in the other neuroactive steroids assessed (i.e., PREG, PROG, DHP, ISOALLO, DHEA, T, DHT and 17β-E) were observed, even if a tendency to towards a decrease that did not reach the statistical significance was reported in the case of T in GF male mice.

### 2.2. Assessment of Neuroactive Steroid Levels in Brain Areas

#### 2.2.1. Hippocampus

**Figure 2:**
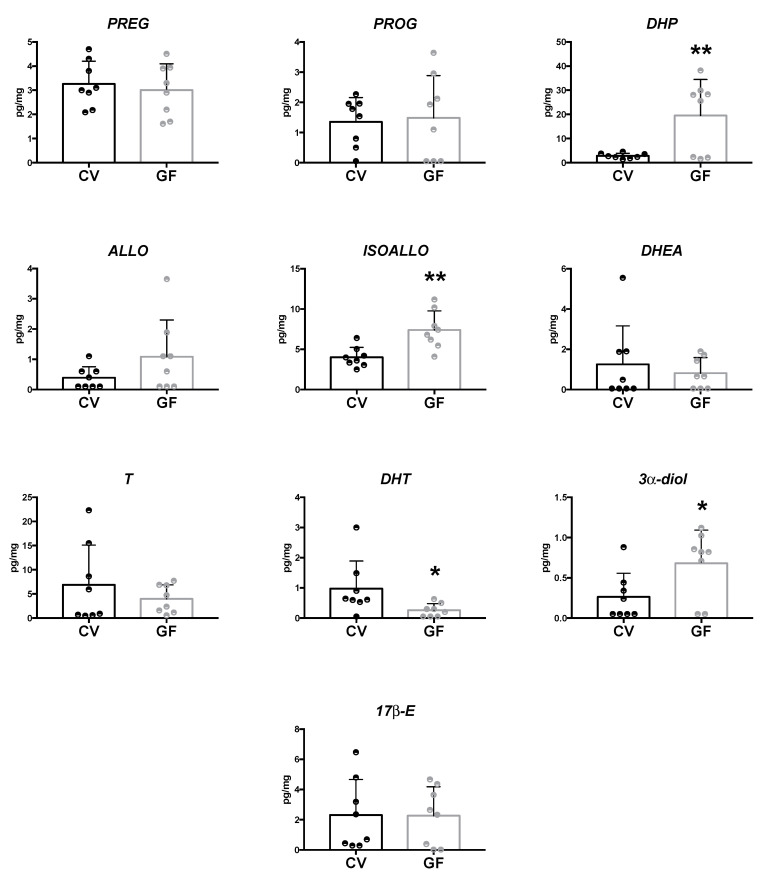
Levels of neuroactive steroids in the hippocampus of germ-free (GF) and conventional (CV) male mice. Data are expressed as pg/mg ± SD, n = 8 for each group. Unpaired student’s *t*-test analysis: * *p* < 0.05 ** *p* < 0.01 vs. CV mice.

In the hippocampus, LC–MS/MS analysis evidenced a significant increase of DHP (*p* = 0.007), ISOALLO (*p =* 0.003), and 3α-diol (*p =* 0.033) levels in GF compared to CV mice (Figure 2). In the same brain region, DHT concentrations were found to be significantly reduced (*p* = 0.049) in GF compared to CV mice (Figure 2). Similar to what was noted in the plasma of GF animals, the levels of PREG, PROG, DHEA, T and 17β-E present in the hippocampus of GF animals were not significantly different vs. those reported in CV animals (Figure 2). Some tendencies towards a decrease (i.e., DHEA and T) and towards an increase (i.e., ALLO) that did not reach statistical significance were reported in GF male mice (Figure 2).

#### 2.2.2. Cerebellum and Cerebral Cortex

**Figure 3:**
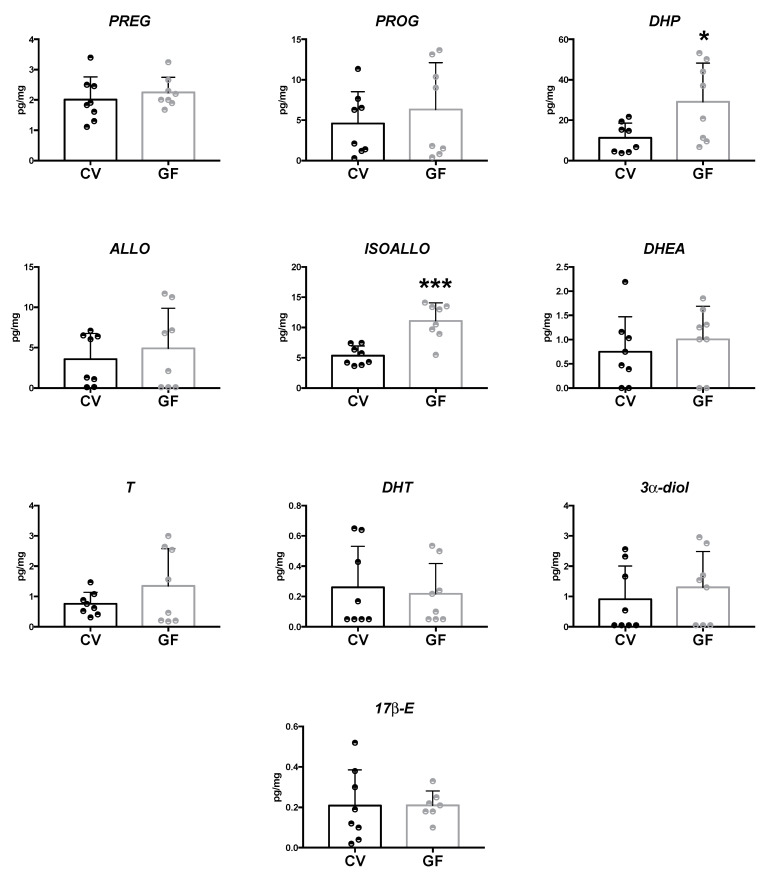
Levels of neuroactive steroids in the cerebellum of germ-free (GF) and conventional (CV) male mice. Data are expressed as pg/mg ± SD, n = 8 for each group. Unpaired student’s *t*-test analysis: * *p* < 0.05 *** *p* < 0.001 vs. CV mice.

**Figure 4:**
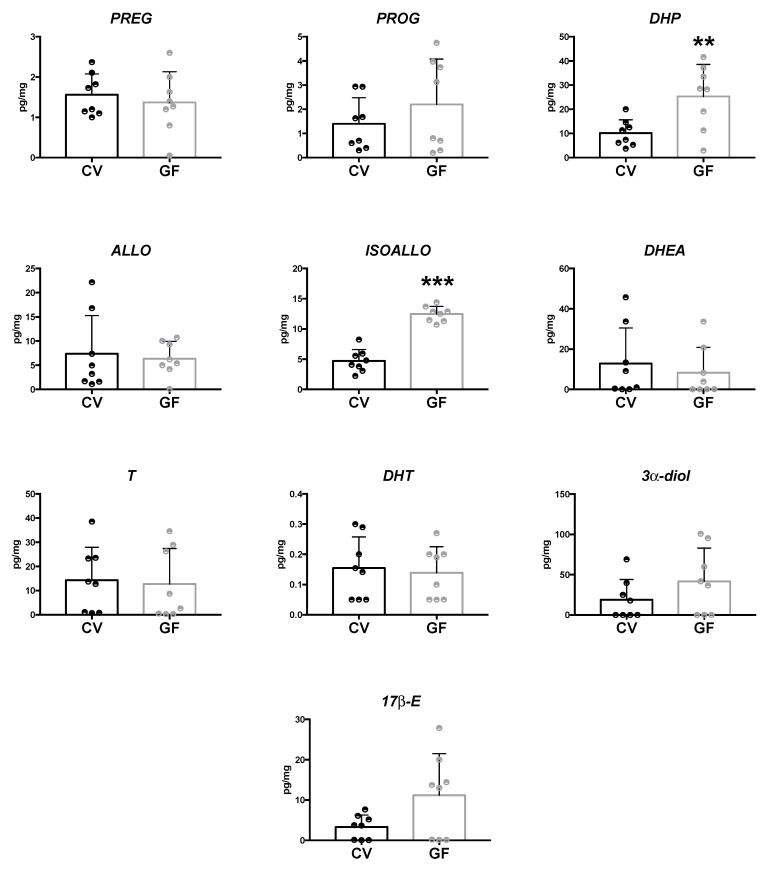
Levels of neuroactive steroids in the cerebral cortex of germ-free (GF) and conventional (CV) male mice. Data are expressed as pg/mg ± SD, n = 8 for each group. Unpaired student’s *t*-test analysis: ** *p* < 0.01 *** *p* < 0.001 vs. CV mice.

The LC–MS/MS analysis of neuroactive steroids showed an increase of DHP and ISOALLO levels in the cerebellum (*p =* 0.028; *p =* 0.0003, respectively; Figure 3) and cerebral cortex (*p =* 0.010; *p* < 0.0001, respectively; Figure 4) of GF compared with CV mice. However, in these two brain areas, the concentrations of the other neuroactive steroids were comparable between the GF and CV mice. Even if a tendency towards an increase was reported in the cerebral cortex of GF male mice for 17β-E and 3α-diol, this did not reach statistical significance.

#### 2.2.3. Hypothalamus

**Figure 5:**
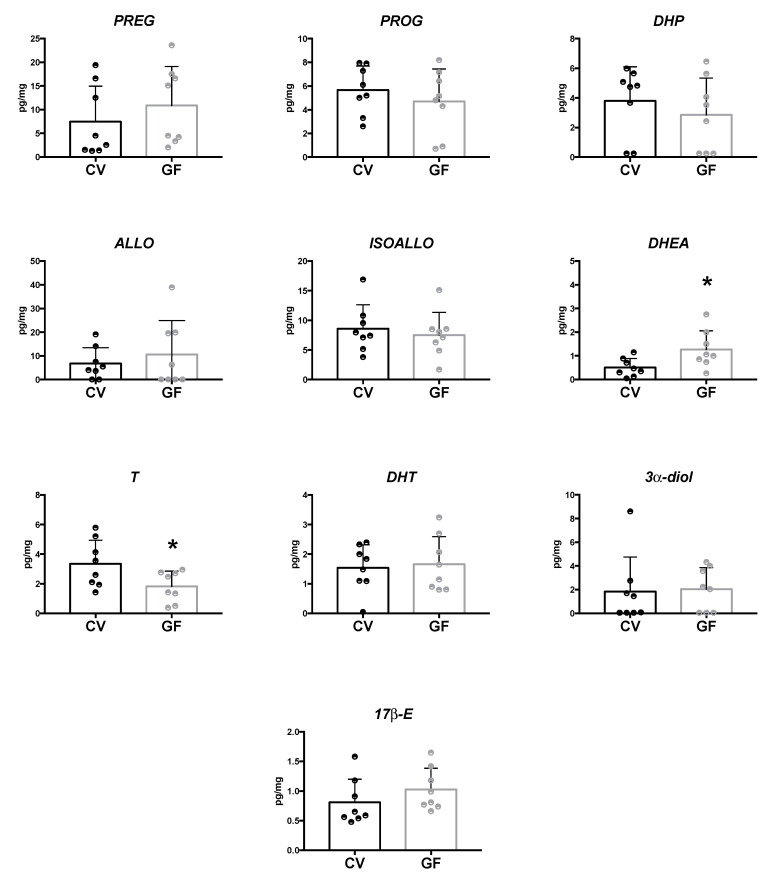
Levels of neuroactive steroids in the hypothalamus of germ-free (GF) and conventional (CV) male mice. Data are expressed as pg/mg ± SD, n = 8 for each group. Unpaired student’s *t*-test analysis: * *p* < 0.05 vs. CV mice.

As showed in Figure 5, in the hypothalamus of GF animals, the LC–MS/MS analysis revealed a significant increase in DHEA concentration (*p =* 0.027), whereas the levels of T were found to be significantly reduced (*p =* 0.040). The concentrations of the other neuroactive steroids assessed were similar in both the GF and CV mice (Figure 5). In the cases of PREG and ALLO, a tendency towards an increase was reported in the hypothalamus of GF male mice that, however, did not reach statistical significance.

## 3. Discussion

The complexity of the bidirectional communication between the gut microbiota, the GI system, and the brain mediated by the multiple signaling pathways and mechanisms of the gut–brain axis is beginning to be elucidated. One of the key pathways in this system is represented by the host–microbe interactions. The possibility of having animals raised without any microorganisms facilitates the investigation of the physiological units and biochemical processes by which such microbes finely tune the development and function of the GI tract and the brain. This study shows for the first time that in the absence of the microbiome, as in GF mice, the concentration of several neuroactive steroids both in plasma and in the brain is altered compared with that of conventionally colonized mice. Our findings highlight a critical role of the microbiome in modulating these important physiological regulators of the nervous system, supporting the existence of a microbial-neuroendocrine signature in preserving brain integrity and function.

Interestingly, two main aspects emerged from these observations. Firstly, the gut microbiota influenced both plasma and CNS levels of neuroactive steroids, but these two compartments appeared to be differentially influenced, as the alterations noted in the plasma differed from those occurring in the brain. Thus, this finding suggests a divergent impact of the gut microbiota on peripheral steroidogenesis and neurosteroidogenesis. This is in line with observations reported in several physiopathological experimental models indicating the divergence of these two pools of steroids [21,39,40].

Secondly, in certain brain regions of GF animals, namely the hippocampus, cerebellum and cerebral cortex, the levels of the same neuroactive steroids were similarly altered. Indeed, in these three brain regions an increase in DHP and its metabolite, ISOALLO, was noted. This finding suggests that these two neuroactive steroids may represent a common signal for several brain areas in the MGBA.

On the other hand, depending on the brain areas considered, specific changes in neuroactive steroids also occurred. Indeed, in the hippocampus of GF mice a decrease in DHT and an increase in 3α-diol levels were observed, while in the hypothalamus, we reported an increase in DHEA and a decrease in T. Thus, there is not only a common pattern of changes (i.e., altered DHP and ISOALLO levels), but also specific changes of neuroactive steroid levels depending on the brain regions considered. DHEA and T levels have been reported to be altered in the hypothalamus of SPF animals as well; however, in this case an opposite pattern was observed [35]. Indeed, in SPF animals, a decrease in DHEA and an increase in T was reported [35], suggesting that different microbiota populations evoked different effects on the levels of these neuroactive steroids. In this context, it is important to highlight that in the hypothalamus of SPF male animals, DHEA levels were positively correlated with *Calditrichaeota* phylum [35].

All these neuroactive steroids exert a variety of physiological effects on the nervous system [39,40]. In this context, it is important to highlight that neuroactive steroids interact with different receptors. Indeed, while DHP—like its precursor PROG—is able to bind with the PROG receptor, ISOALLO—much like ALLO (i.e., another metabolite of PROG)—interacts with the GABA-A receptor. However, ISOALLO, in contrast to ALLO— which is a potent ligand of the GABA-A receptor [41,42]—does not bind directly to this neurotransmitter receptor [43], but instead antagonizes the effect of ALLO on the GABA-A receptor [44,45]. In addition, T and DHT, even if with different affinities, bind to the androgen receptor (AR), while their metabolite 3α-diol interacts with the GABA-A receptor. The mechanism of action of DHEA in the nervous system has not yet been fully characterized. Observations so far obtained show modulatory effects of this neuroactive steroid on membrane receptors, such as GABA-A, NMDA and sigma 1 receptors [46,47,48,49], while others suggest interactions with AR and its upregulation [50,51,52,53]. In this context, it is interesting to note that gut microbiota disturbances alter the expression of GABA-A [54,55] and NMDA receptors [56] in the rodent brain.

A further interesting link in the context of the gut microbiota–brain axis may be provided by the finding that chronic treatment with finasteride (i.e., an inhibitor of steroidogenic enzyme 5alpha-reductase that converts PROG and T into their metabolites) induced changes in the gut microbiota populations of male rats (i.e., an increase in *Bacteroidetes* phylum and in the *Prevotellaceae* family) [57] and in post-finasteride patients [58]. In addition, similarly to what we have reported in the cerebellum of GF animals, an increase in DHP and ISOALLO also occurred in the cerebellum of finasteride-treated rats [59]. A possible hypothesis for this increase in the cerebellum of GF animals could be an increase in the gene expression of the enzyme 5alpha-reductase, as we previously reported in finasteride-treated rats [59].

Altogether, these observations show for the first time that also neuroactive steroids in brain areas, like steroid hormones in the periphery [17,18,19,20], are affected by the gut microbiota population. Therefore, these findings may suggest that important physiological regulators of nervous function, such as neuroactive steroids, represent another molecular signal in the context of the MGBA and potential markers of MGBA-dependent psychiatric disorders. Future experiments will be needed to consolidate this important link and to evaluate the biomolecular mechanisms involved.

## 4. Materials and Methods

### 4.1. Animals

C57/Bl6 mice were purchased from Taconic (Hudson, NY, USA), and were bred as GF or conventionally raised in the animal facility of the Bioscience building, University College Cork, Cork Ireland. Breeding was performed according to supplier guidelines, and we used male offspring from F1-generation. GF male mice were housed 4 per cage in individually ventilated cages (area: 420 cm^2^. Arrowmight, UK), sex- and age-matched conventional mice were housed 4 mice/cage but in standard cages (area: 330 cm^2^. NKP isotech, UK). GF and conventional mice were kept at the same temperature (21 ± 1 °C) and humidity (55–60%) conditions on a 12 h light/dark cycle and maintained on an ad libitum autoclaved water and autoclaved diet (pellet, Special Diet Services, Product code 801010).

### 4.2. Tissue Collection

Male GF (12 ±1 weeks old; n = 8) and age- and sex- matched conventional mice (n = 8) were culled by decapitation and trunk blood was immediately collected in a K2 EDTA lavender-top vacutainer (BD Life Sciences). Blood was centrifuged at 3500× *g* for 15 min at 4 °C, and plasma was collected in pre-weighed 1.5 Eppendorf tubes and stored at −80 °C until analysis. Brains were immediately collected and placed in petri dished containing wet ice. For each brain, regions such as the hypothalamus, hippocampus, cerebellum, or cerebral cortex were manually dissected and placed in pre-weighed 1.5 Eppendorf tubes. Brain regions were snap frozen at −80 °C and kept in these conditions until analysis.

### 4.3. Reagents and Chemicals

Pregnenolone (PREG), pregnenolone-20,21-^13^C_2_-16,16 D_2_ (^13^C_2_ D_2_–PREG), progesterone (PROG), progesterone-2,3,4,20,25-^13^C_5_ (^13^C_5_–PROG), 17β-Estradiol (17β-E), 17β-Estradiol-2,3,4-^13^C_3_ (^13^C_3_-17β-E) dihydroprogesterone (DHP), allopregnanolone (ALLO), isoallopregnanolone (ISOALLO), testosterone (T), dihydrotestosterone (DHT), 5α-androstane-3α,17β-diol (3α-diol) and dehydroepiandrosterone (DHEA) were purchased from Merck Life Science, Italy. Acetonitrile, acetic acid, formic acid, methanol, 2-propanol and water were HPLC grade (Merck Life Science, Milano, Italy).

### 4.4. Liquid Chromatography–Tandem Mass Spectrometry Analysis

For the quantitative analysis of neuroactive steroids, brain tissues and plasma samples were extracted and purified as previously described [21,60,61,62]. ^13^C_3_-17β-E (2 ng/sample), ^13^C_5_–PROG (0.4 ng/sample) and ^13^C_2_ D_2_–PREG (10 ng/sample) were used as internal standards. For quantitative analysis of steroids, cerebral cortex, hypothalamus, hippocampus, cerebellum and plasma were collected, and internal standards were added. Tissue samples were homogenized using a Tissue Lyser (Qiagen, Italy), in ice-cold MeOH/acetic acid 1%. All tissues and plasma were purified by organic phase extraction, as previously described [21,60,61,62]. The analysis was conducted by liquid chromatography (LC) using an LC Pump Plus and Surveyor Autosampler Plus (Thermo Fisher Scientific, San Jose, CA, USA) with a linear ion trap-mass spectrometer (LTQ, Thermo Fisher Scientific, San Jose, CA, USA) operating in positive atmospheric pressure chemical ionization (APCI+). The chromatographic separation was achieved with a Hypersil Gold column C18 (100 × 2.1 mm, 3 μm; ThermoFisher Scientific) maintained at 40 °C. The mobile phases consisted of 0.1% formic acid in water (mobile phase A) and 0,1% formic acid in methanol (mobile phase B). The gradient elution was as follows: 0–1.50 min 70% A, 30% B; 1.50–2.00 min 55% A, 45%B; 2.00–3.00 min. 55% A, 45% B; 3.00–35.00 min. linear gradient to 36% A, 64% B; 35.00–40.00 min. 25% A, 75% B; 41.00–45.00 min. 1% A, 99% B; 45.00–45.20 min. 70% A, 30% B and 45.40–55.00 min equilibrated with 70% A and 30% B. A sample of 25 μL was injected at a flowrate of 0.250 mL/min. The divert valve was set at 0–8 min to waste, 8–45 min to source and 45–55 min to waste. The injector needle was washed with MeOH/Water 1/1 (*v/v*). Quantitative analysis was performed on the basis of calibration curves prepared and analyzed using standards. LC–MS/MS peaks were appraised using the software Excalibur^®^ release 2.0 SR2 (Thermo Fisher Scientific, San Jose, CA, USA). Quantitative analysis of all steroids was achieved based on freshly prepared calibration curves. Detection limits were 0.02 pg/μL or pg/mg for T and 17β-E, 0.05 pg/μL or pg/mg for PREG, PROG, 3α-diol, DHEA, DHT; 0.1 pg/μL or pg/mg for ALLO and ISOALLO; 0.25 pg/μL or pg/mg for DHP.

### 4.5. Statistical Analysis

Data for LC–MS/MS (n = 8 per experimental group) were analyzed by unpaired Student’s t-test, after checking for normal distribution with the Kolmogorov–Smirnov test. *p* < 0.05 was considered significant. Analyses were performed using Prism, version 7.0a (GraphPad Software Inc., San Diego, CA, USA).

## References

[1] Cryan J.F., O’Riordan K.J., Cowan C.S.M., Sandhu K.V., Bastiaanssen T.F.S., Boehme M., Codagnone M.G., Cussotto S., Fulling C., Golubeva A.V. (2019). The Microbiota-Gut-Brain Axis. Physiol. Rev..

[2] Dalile B., Van Oudenhove L., Vervliet B., Verbeke K. (2019). The role of short-chain fatty acids in microbiota-gut-brain communication. Nat. Rev. Gastroenterol. Hepatol..

[3] Lai Y., Liu C.W., Yang Y., Hsiao Y.C., Ru H., Lu K. (2021). High-coverage metabolomics uncovers microbiota-driven biochemical landscape of interorgan transport and gut-brain communication in mice. Nat. Commun..

[4] Cussotto S., Sandhu K.V., Dinan T.G., Cryan J.F. (2018). The Neuroendocrinology of the Microbiota-Gut-Brain Axis: A Behavioural Perspective. Front. Neuroendocr..

[5] Mukherji A., Kobiita A., Ye T., Chambon P. (2013). Homeostasis in intestinal epithelium is orchestrated by the circadian clock and microbiota cues transduced by TLRs. Cell.

[6] Org E., Mehrabian M., Parks B.W., Shipkova P., Liu X., Drake T.A., Lusis A.J. (2016). Sex differences and hormonal effects on gut microbiota composition in mice. Gut Microbes.

[7] Jasarevic E., Morrison K.E., Bale T.L. (2016). Sex differences in the gut microbiome-brain axis across the lifespan. Philos. Trans. R. Soc. B Biol. Sci..

[8] Fields C.T., Chassaing B., Paul M.J., Gewirtz A.T., de Vries G.J. (2017). Vasopressin deletion is associated with sex-specific shifts in the gut microbiome. Gut Microbes.

[9] Yurkovetskiy L., Burrows M., Khan A.A., Graham L., Volchkov P., Becker L., Antonopoulos D., Umesaki Y., Chervonsky A.V. (2013). Gender bias in autoimmunity is influenced by microbiota. Immunity.

[10] Moreno-Indias I., Sanchez-Alcoholado L., Sanchez-Garrido M.A., Martin-Nunez G.M., Perez-Jimenez F., Tena-Sempere M., Tinahones F.J., Queipo-Ortuno M.I. (2016). Neonatal androgen exposure causes persistent gut microbiota dysbiosis related to metabolic disease in adult female rats. Endocrinology.

[11] Menon R., Watson S.E., Thomas L.N., Allred C.D., Dabney A., Azcarate-Peril M.A., Sturino J.M. (2013). Diet complexity and estrogen receptor beta status affect the composition of the murine intestinal microbiota. Appl. Environ. Microbiol..

[12] Santos-Marcos J.A., Barroso A., Rangel-Zuniga O.A., Perdices-Lopez C., Haro C., Sanchez-Garrido M.A., Molina-Abril H., Ohlsson C., Perez-Martinez P., Poutanen M. (2020). Interplay between gonadal hormones and postnatal overfeeding in defining sex-dependent differences in gut microbiota architecture. Aging.

[13] Tramullas M., Collins J.M., Fitzgerald P., Dinan T.G., SM O.M., Cryan J.F. (2021). Estrous cycle and ovariectomy-induced changes in visceral pain are microbiota-dependent. iScience.

[14] Mueller S., Saunier K., Hanisch C., Norin E., Alm L., Midtvedt T., Cresci A., Silvi S., Orpianesi C., Verdenelli M.C. (2006). Differences in fecal microbiota in different European study populations in relation to age, gender, and country: A cross-sectional study. Appl. Environ. Microbiol..

[15] Dominianni C., Sinha R., Goedert J.J., Pei Z., Yang L., Hayes R.B., Ahn J. (2015). Sex, body mass index, and dietary fiber intake influence the human gut microbiome. PLoS ONE.

[16] Haro C., Rangel-Zuniga O.A., Alcala-Diaz J.F., Gomez-Delgado F., Perez-Martinez P., Delgado-Lista J., Quintana-Navarro G.M., Landa B.B., Navas-Cortes J.A., Tena-Sempere M. (2016). Intestinal Microbiota Is Influenced by Gender and Body Mass Index. PLoS ONE.

[17] Clarke G., Stilling R.M., Kennedy P.J., Stanton C., Cryan J.F., Dinan T.G. (2014). Minireview: Gut microbiota: The neglected endocrine organ. Mol. Endocrinol..

[18] Fuhrman B.J., Feigelson H.S., Flores R., Gail M.H., Xu X., Ravel J., Goedert J.J. (2014). Associations of the fecal microbiome with urinary estrogens and estrogen metabolites in postmenopausal women. J. Clin. Endocrinol. Metab..

[19] Goedert J.J., Jones G., Hua X., Xu X., Yu G., Flores R., Falk R.T., Gail M.H., Shi J., Ravel J. (2015). Investigation of the association between the fecal microbiota and breast cancer in postmenopausal women: A population-based case-control pilot study. J. Natl. Cancer Inst..

[20] Markle J.G., Frank D.N., Mortin-Toth S., Robertson C.E., Feazel L.M., Rolle-Kampczyk U., von Bergen M., McCoy K.D., Macpherson A.J., Danska J.S. (2013). Sex differences in the gut microbiome drive hormone-dependent regulation of autoimmunity. Science.

[21] Caruso D., Pesaresi M., Abbiati F., Calabrese D., Giatti S., Garcia-Segura L.M., Melcangi R.C. (2013). Comparison of plasma and cerebrospinal fluid levels of neuroactive steroids with their brain, spinal cord and peripheral nerve levels in male and female rats. Psychoneuroendocrinology.

[22] Giatti S., Diviccaro S., Serafini M.M., Caruso D., Garcia-Segura L.M., Viviani B., Melcangi R.C. (2019). Sex differences in steroid levels and steroidogenesis in the nervous system: Physiopathological role. Front. Neuroendocrinol..

[23] Melcangi R.C., Garcia-Segura L.M., Mensah-Nyagan A.G. (2008). Neuroactive steroids: State of the art and new perspectives. Cell Mol. Life Sci..

[24] Giatti S., Diviccaro S., Falvo E., Garcia-Segura L.M., Melcangi R.C. (2020). Physiopathological role of the enzymatic complex 5alpha-reductase and 3alpha/beta-hydroxysteroid oxidoreductase in the generation of progesterone and testosterone neuroactive metabolites. Front. Neuroendocrinol..

[25] Balleza D., Sacchi M., Vena G., Galloni D., Puia G., Facci P., Alessandrini A. (2015). Effects of neurosteroids on a model membrane including cholesterol: A micropipette aspiration study. Biochim. Biophys. Acta.

[26] Sacchi M., Balleza D., Vena G., Puia G., Facci P., Alessandrini A. (2015). Effect of neurosteroids on a model lipid bilayer including cholesterol: An Atomic Force Microscopy study. Biochim. Biophys. Acta.

[27] Brandt N., Vierk R., Fester L., Anstotz M., Zhou L., Heilmann L.F., Kind S., Steffen P., Rune G.M. (2020). Sex-specific Difference of Hippocampal Synaptic Plasticity in Response to Sex Neurosteroids. Cereb. Cortex.

[28] Islam M.N., Sakimoto Y., Jahan M.R., Ishida M., Tarif A.M.M., Nozaki K., Masumoto K.H., Yanai A., Mitsushima D., Shinoda K. (2020). Androgen Affects the Dynamics of Intrinsic Plasticity of Pyramidal Neurons in the CA1 Hippocampal Subfield in Adolescent Male Rats. Neuroscience.

[29] Frye C.A. (2007). Some rewarding effects of androgens may be mediated by actions of its 5alpha-reduced metabolite 3alpha-androstanediol. Pharm. Biochem. Behav..

[30] Rosellini R.A., Svare B.B., Rhodes M.E., Frye C.A. (2001). The testosterone metabolite and neurosteroid 3alpha-androstanediol may mediate the effects of testosterone on conditioned place preference. Brain Res. Brain Res. Rev..

[31] Compagnone N.A., Mellon S.H. (1998). Dehydroepiandrosterone: A potential signalling molecule for neocortical organization during development. Proc. Natl. Acad. Sci. USA.

[32] Karishma K.K., Herbert J. (2002). Dehydroepiandrosterone (DHEA) stimulates neurogenesis in the hippocampus of the rat, promotes survival of newly formed neurons and prevents corticosterone-induced suppression. Eur. J. Neurosci..

[33] Zhang L., Li B., Ma W., Barker J.L., Chang Y.H., Zhao W., Rubinow D.R. (2002). Dehydroepiandrosterone (DHEA) and its sulfated derivative (DHEAS) regulate apoptosis during neurogenesis by triggering the Akt signaling pathway in opposing ways. Brain Res. Mol. Brain Res..

[34] Perez-Neri I., Montes S., Ojeda-Lopez C., Ramirez-Bermudez J., Rios C. (2008). Modulation of neurotransmitter systems by dehydroepiandrosterone and dehydroepiandrosterone sulfate: Mechanism of action and relevance to psychiatric disorders. Prog. Neuropsychopharmacol. Biol. Psychiatry.

[35] Chu L., Huang Y., Xu Y., Wang L.K., Lu Q. (2021). An LC-APCI(+)-MS/MS-based method for determining the concentration of neurosteroids in the brain of male mice with different gut microbiota. J. Neurosci. Methods.

[36] Scott G.A., Terstege D.J., Vu A.P., Law S., Evans A., Epp J.R. (2020). Disrupted Neurogenesis in Germ-Free Mice: Effects of Age and Sex. Front. Cell Dev. Biol..

[37] Hoban A.E., Stilling R.M.G., Moloney R.D., Shanahan F., Dinan T.G., Cryan J.F., Clarke G. (2017). Microbial regulation of microRNA expression in the amygdala and prefrontal cortex. Microbiome.

[38] Neufeld K.A., Kang N., Bienenstock J., Foster J.A. (2011). Effects of intestinal microbiota on anxiety-like behavior. Commun. Integr. Biol..

[39] Melcangi R.C., Giatti S., Calabrese D., Pesaresi M., Cermenati G., Mitro N., Viviani B., Garcia-Segura L.M., Caruso D. (2014). Levels and actions of progesterone and its metabolites in the nervous system during physiological and pathological conditions. Prog. Neurobiol..

[40] Melcangi R.C., Giatti S., Garcia-Segura L.M. (2016). Levels and actions of neuroactive steroids in the nervous system under physiological and pathological conditions: Sex-specific features. Neurosci. Biobehav. Rev..

[41] Lambert J.J., Belelli D., Peden D.R., Vardy A.W., Peters J.A. (2003). Neurosteroid modulation of GABAA receptors. Prog. Neurobiol..

[42] Belelli D., Lambert J.J. (2005). Neurosteroids: Endogenous regulators of the GABA(A) receptor. Nat. Rev. Neurosci..

[43] Bitran D., Hilvers R.J., Kellogg C.K. (1991). Anxiolytic effects of 3 alpha-hydroxy-5 alpha[beta]-pregnan-20-one: Endogenous metabolites of progesterone that are active at the GABAA receptor. Brain Res..

[44] Wang M., He Y., Eisenman L.N., Fields C., Zeng C.M., Mathews J., Benz A., Fu T., Zorumski E., Steinbach J.H. (2002). 3beta -hydroxypregnane steroids are pregnenolone sulfate-like GABA(A) receptor antagonists. J. Neurosci..

[45] Backstrom T., Wahlstrom G., Wahlstrom K., Zhu D., Wang M.D. (2005). Isoallopregnanolone; an antagonist to the anaesthetic effect of allopregnanolone in male rats. Eur. J. Pharm..

[46] Monnet F.P., Mahe V., Robel P., Baulieu E.E. (1995). Neurosteroids, via sigma receptors, modulate the [3H] norepinephrine release evoked by N-methyl-D-aspartate in the rat hippocampus. Proc. Natl. Acad. Sci. USA.

[47] Demirgoren S., Majewska M.D., Spivak C.E., London E.D. (1991). Receptor binding and electrophysiological effects of dehydroepiandrosterone sulfate, an antagonist of the GABAA receptor. Neuroscience.

[48] Bergeron R., de Montigny C., Debonnel G. (1996). Potentiation of neuronal NMDA response induced by dehydroepiandrosterone and its suppression by progesterone: Effects mediated via sigma receptors. J. Neurosci..

[49] Mehta A.K., Ticku M.K. (1999). An update on GABAA receptors. Brain Res. Brain Res. Rev..

[50] Mo Q., Lu S., Garippa C., Brownstein M.J., Simon N.G. (2009). Genome-wide analysis of DHEA- and DHT-induced gene expression in mouse hypothalamus and hippocampus. J. Steroid. Biochem. Mol. Biol..

[51] Mo Q., Lu S.F., Simon N.G. (2006). Dehydroepiandrosterone and its metabolites: Differential effects on androgen receptor trafficking and transcriptional activity. J. Steroid Biochem. Mol. Biol..

[52] Mo Q., Lu S.F., Hu S., Simon N.G. (2004). DHEA and DHEA sulfate differentially regulate neural androgen receptor and its transcriptional activity. Brain Res. Mol. Brain Res..

[53] Lu S.F., Mo Q., Hu S., Garippa C., Simon N.G. (2003). Dehydroepiandrosterone upregulates neural androgen receptor level and transcriptional activity. J. Neurobiol..

[54] Liang L., Zhou H., Zhang S., Yuan J., Wu H. (2017). Effects of gut microbiota disturbance induced in early life on the expression of extrasynaptic GABA-A receptor alpha5 and delta subunits in the hippocampus of adult rats. Brain Res. Bull..

[55] Bravo J.A., Forsythe P., Chew M.V., Escaravage E., Savignac H.M., Dinan T.G., Bienenstock J., Cryan J.F. (2011). Ingestion of Lactobacillus strain regulates emotional behavior and central GABA receptor expression in a mouse via the vagus nerve. Proc. Natl. Acad. Sci. USA.

[56] Maqsood R., Stone T.W. (2016). The Gut-Brain Axis, BDNF, NMDA and CNS Disorders. Neurochem. Res..

[57] Diviccaro S., Giatti S., Borgo F., Barcella M., Borghi E., Trejo J.L., Garcia-Segura L.M., Melcangi R.C. (2019). Treatment of male rats with finasteride, an inhibitor of 5alpha-reductase enzyme, induces long-lasting effects on depressive-like behavior, hippocampal neurogenesis, neuroinflammation and gut microbiota composition. Psychoneuroendocrinology.

[58] Borgo F., Macandog A.D., Diviccaro S., Falvo E., Giatti S., Cavaletti G., Melcangi R.C. (2020). Alterations of gut microbiota composition in post-finasteride patients: A pilot study. J. Endocrinol. Investig..

[59] Giatti S., Foglio B., Romano S., Pesaresi M., Panzica G., Garcia-Segura L.M., Caruso D., Melcangi R.C. (2016). Effects of Subchronic Finasteride Treatment and Withdrawal on Neuroactive Steroid Levels and their Receptors in the Male Rat Brain. Neuroendocrinology.

[60] Pesaresi M., Maschi O., Giatti S., Garcia-Segura L.M., Caruso D., Melcangi R.C. (2010). Sex differences in neuroactive steroid levels in the nervous system of diabetic and non-diabetic rats. Horm. Behav..

[61] Caruso D., Scurati S., Maschi O., De Angelis L., Roglio I., Giatti S., Garcia-Segura L.M., Melcangi R.C. (2008). Evaluation of neuroactive steroid levels by liquid chromatography-tandem mass spectrometry in central and peripheral nervous system: Effect of diabetes. Neurochem. Int..

[62] Caruso D., Pesaresi M., Maschi O., Giatti S., Garcia-Segura L.M., Melcangi R.C. (2010). Effect of short-and long-term gonadectomy on neuroactive steroid levels in the central and peripheral nervous system of male and female rats. J. Neuroendocrinol..

